# Determinants of final height in X-linked hypophosphatemia: impact of diagnostic delay and baseline growth in a Brazilian cohort

**DOI:** 10.3389/fped.2026.1845607

**Published:** 2026-07-02

**Authors:** Mauro Borghi, João Pedro Borghi Moreira, Leopoldo Muniz da Silva

**Affiliations:** 1 Faculdade de Ciências Médicas da Santa Casa de São Paulo (FCMSCSP), São Paulo, SP, Brazil; 2 Unidade de Endocrinologia Pediátrica, Ambulatório de Metabolismo Ósseo, Irmandade da Santa Casa de Misericórdia de São Paulo (ISCMSP), São Paulo, SP, Brazil; 3 Faculdade de Medicina, Universidade de Santo Amaro (UNISA), São Paulo, SP, Brazil; 4 Rede D’Or, D’Or Institute for Research and Education (IDOR), São Paulo, SP, Brazil

**Keywords:** genetics, height, pediatrics, rickets, x-linked hypophosphatemia

## Abstract

**Background:**

X-linked hypophosphatemia (XLH) is a rare genetic disorder characterized by impaired phosphate metabolism, leading to rickets and growth failure. Delayed diagnosis may worsen growth outcomes, particularly in settings with limited access to specialized care. This study evaluated determinants of final height in a Brazilian cohort, focusing on age at diagnosis and baseline growth status.

**Methods:**

This retrospective observational cohort study included 41 patients with molecularly confirmed XLH followed at a tertiary referral center in Brazil between 1971 and 2025. Anthropometric and clinical data were extracted from medical records. Final height analysis was restricted to 20 patients treated exclusively with conventional therapy. Correlations were assessed using Spearman coefficients, and multivariable linear regression was performed to identify independent predictors of final height Z score.

**Results:**

At diagnosis, the mean height-for-age Z-score was −1.90 ± 1.56, indicating significant baseline growth impairment. Age at diagnosis was inversely correlated with height-for-age Z-score (*r* = −0.77; *P* = 0.02), while no correlation was observed with BMI-for-age Z-score. Among patients who reached final height, the mean final height Z-score was −2.96 ± 1.15. In multivariable analysis, height-for-age Z-score at diagnosis (*β* = 0.10; 95% CI 0.04 to 0.15; *P* = 0.009), age at diagnosis (*β* = −0.04; 95% CI −0.07 to −0.02; *P* = 0.007), and target height Z-score (*β* = 0.37; 95% CI 0.15 to 0.59; *P* = 0.01) were independently associated with final height. Sex and BMI-for-age Z-score were not significantly associated with the outcome.

**Conclusions:**

Final height in XLH is influenced by a combination of genetic potential, baseline growth impairment, and timing of diagnosis. Delayed diagnosis is associated with greater growth deficits and suboptimal adult height, even under conventional therapy. These findings highlight the importance of early recognition and provide a benchmark for evaluating growth outcomes in the era of targeted therapies.

## Introduction

X-linked hypophosphatemia (XLH) is the most common inherited form of hypophosphatemic rickets, with an estimated incidence of approximately 3.9 per 100,000 live births (≈1 in 25,000) (1). The disorder is caused by loss-of-function variants in the *PHEX* gene, leading to increased circulating levels of fibroblast growth factor 23 (FGF23). Excess FGF23 reduces renal tubular phosphate reabsorption and suppresses 1*α*-hydroxylation of vitamin D, resulting in chronic hypophosphatemia, inappropriately low or normal levels of 1,25-dihydroxyvitamin D, and impaired skeletal mineralization (1, 2). Clinically, XLH is characterized by progressive lower-limb deformities, bone pain, dental abnormalities including recurrent abscesses, and disproportionate short stature (1, 3).

The phenotypic spectrum of XLH is highly heterogeneous, even among individuals harboring identical *PHEX* variants. This variability reflects the X-linked dominant pattern of inheritance and, in females, the modulatory effect of X chromosome inactivation (2, 4). Consistent with these mechanisms, recent data, including Brazilian cohorts, have demonstrated a lack of robust correlation between genotype and phenotype, indicating that clinical severity cannot be reliably predicted from the underlying genetic variant alone (5, 6). Although females are more frequently affected, males, who are hemizygous for *PHEX*, typically exhibit more severe skeletal manifestations (4).

Short stature is a prominent clinical feature of XLH and typically begins in early childhood, often with disproportionate growth related to progressive lower limb deformities (7). In addition to its physical manifestations, impaired linear growth has been associated with reduced health-related quality of life, including effects on self-perception, social participation, and long-term functional outcomes (8). Early diagnosis is therefore critical to limit progression of skeletal deformities and to optimize growth trajectories (1, 9).

Conventional therapy with oral phosphate and active vitamin D analogues partially corrects biochemical abnormalities and improves rickets but rarely normalizes serum phosphate levels and is associated with incomplete catch-up growth (10). The role of adjunctive growth hormone remains uncertain (10, 11). Since 2018, burosumab, a monoclonal antibody targeting FGF23, has transformed disease management, demonstrating superior effects on phosphate homeostasis, skeletal outcomes, and linear growth in children (12, 13). Despite these advances, delayed diagnosis remains common in Brazil and is associated with greater initial height deficits and increased orthopedic burden (5). Long-term real-world data from Brazilian cohorts remain limited, particularly regarding final height and the impact of early diagnosis.

This study evaluates clinical and anthropometric characteristics at diagnosis, final height outcomes, and factors associated with final height in a molecularly confirmed Brazilian cohort. Analyses of final height were restricted to patients treated exclusively with conventional therapy, with particular focus on the influence of age at diagnosis and baseline height deficit.

## Methods

### Study design and ethical considerations

This retrospective observational cohort study included patients with confirmed XLH followed at the Bone Metabolism Outpatient Clinic of the Irmandade da Santa Casa de Misericórdia de São Paulo, Brazil, between 1971 and 2025. All participants or their legal guardians provided written informed consent. The study was approved by the institutional research ethics committee (CEP ISCMSP; protocol 75/04; CAAE 3.225.914) and conducted in accordance with the Declaration of Helsinki and Brazilian National Health Council Resolution 466/2012.

Patients were eligible if they presented with progressive lower limb bowing beginning after independent ambulation, radiographic evidence of rickets characterized by metaphyseal fraying, epiphyseal widening, or osteopenia, and a biochemical profile consistent with XLH, including chronic hypophosphatemia, reduced tubular maximum reabsorption of phosphate corrected for glomerular filtration rate (TmP/GFR), hyperphosphaturia, and inappropriately normal or low levels of 1,25-dihydroxyvitamin D. All cases required molecular confirmation of a pathogenic or likely pathogenic *PHEX* variant. Patients with clinical suspicion of XLH without molecular confirmation were excluded.

### Clinical data collection, anthropometric assessment, and variable definitions

Demographic, clinical, radiographic, biochemical, and anthropometric data were extracted from medical records. Age at diagnosis was defined as the age at first confirmed clinical and biochemical diagnosis. Height measurements were originally obtained during routine clinical care by trained healthcare professionals using standardized methods and calibrated equipment according to World Health Organization recommendations (14). Recumbent length was measured with an infantometer for children younger than 2 years, whereas standing height was measured with a wall mounted stadiometer for children aged 2 years or older and adults, both with a precision of 1 mm. These measurements were subsequently extracted retrospectively from medical records for the present study. All measurements were transformed into z-scores by comparing them with age- and sex-specific norms for healthy children (15). Familial target height was estimated using the Tanner method and converted to Z-scores based on CDC references (14, 16). Final height was defined as a height velocity <2 cm per year and/or radiological evidence of epiphyseal closure, when available (17).

Conventional therapy consisted of oral phosphate supplementation combined with active vitamin D analogues and was generally initiated shortly after diagnosis according to standard institutional practice. Among patients who reached final height, only those with complete longitudinal anthropometric data and treated exclusively with conventional therapy were included in the final height analysis. Patients exposed to burosumab were excluded from the analysis of final height outcomes. Review of the available medical records did not identify systematic delays between diagnosis and treatment initiation. Although the exact date of treatment initiation was not consistently available for retrospective extraction, conventional therapy was routinely prescribed at the time of diagnosis and, as our institution is a national referral center for metabolic bone disorders, phosphate supplementation and active vitamin D analogues were generally made available immediately after diagnosis.

Orthopedic surgery was recorded as a binary variable. Indications included severe *genu varum* or *valgum*, mechanical axis deviation, and functional impairment. Surgical procedures were performed after 6 years of age and only following metabolic stabilization.

### Molecular genetic analysis

Genomic DNA was extracted from peripheral blood or buccal swab samples using commercially available kits. Targeted next generation sequencing of the *PHEX* gene was performed, covering all coding exons and flanking intronic regions. Sequencing was conducted on an Illumina HiSeq platform, with alignment to the GRCh37/hg19 reference genome. Variant calling was performed using GATK, and copy number variation analysis was conducted using ExomeDepth. Variant classification followed the American College of Medical Genetics and Genomics 2015 guidelines (16), incorporating data from ClinVar, HGMD, gnomAD, and Mastermind, along with in silico prediction tools including REVEL and SpliceAI. Molecular genetic testing was performed as part of routine clinical care during patient follow up. Genetic data were retrospectively retrieved from medical records and institutional databases for the present study.

### Statistical analysis

Given the rarity of XLH, no formal sample size calculation was performed. Continuous variables were summarized as mean ± standard deviation or median (interquartile range), as appropriate, and categorical variables as frequencies and percentages. Normality of data distribution was assessed using the Shapiro–Wilk test. Comparisons between groups were performed using the Student's *t*-test or Mann–Whitney *U*-test for continuous variables, according to data distribution. Correlations between age at diagnosis and baseline anthropometric parameters, as well as between baseline and final height Z-scores and familial target height, were evaluated using Spearman rank correlation coefficients. Scatter plots stratified by sex were generated to visualize the relationships between age at diagnosis and height-for-age Z-score and BMI-for-age Z-score at diagnosis. Locally weighted scatterplot smoothing (LOESS) curves with 95% confidence intervals were superimposed to illustrate trends in the data. To evaluate temporal trends in diagnostic timing, a simple linear regression analysis was performed with age at diagnosis as the dependent variable and year of birth as the independent variable. Regression coefficients with 95% confidence intervals were estimated.

To identify independent predictors of final height Z-score, a multivariable linear regression model was constructed including clinically relevant variables: height-for-age Z-score at diagnosis, age at diagnosis, target height Z-score, sex, and BMI-for-age Z-score. Variables were selected *a priori* based on clinical relevance. Regression coefficients (*β*) with 95% confidence intervals (CI) were reported. Model assumptions, including linearity and homoscedasticity, were assessed through residual analysis. Given the limited sample size, the number of variables included in the multivariable model was restricted to minimize overfitting, following current methodological recommendations (18). A two-sided *P* value < 0.05 was considered statistically significant. Statistical analyses were performed using R software (version 4.3.2; R Foundation for Statistical Computing, Vienna, Austria).

## Results

### Baseline characteristics

A total of 41 patients followed at a single tertiary pediatric unit specializing in inherited metabolic bone disorders were included in this study, all presenting clinical features consistent with XLH. Among these, 63.4% (26/41) were female. The mean age at diagnosis was 49 months, with 72% of patients diagnosed before 5 years of age. Radiographic findings consistent with rickets, including metaphyseal fraying, epiphyseal widening, and osteopenia, were observed in most patients at baseline (87.8%; 36/41). Lower limb deformities confirmed by radiographic evaluation at presentation were observed in 57.7% (24/41) of cases.

Family history data were available for 27 patients, among whom 15 (55.5%) had a positive family history of XLH and 12 (44.5%) had no known affected relatives and were therefore considered index cases. Family history information was unavailable for the remaining 14 patients. No patients demonstrated clinical evidence of hearing loss, severe dental abnormalities, or nephrocalcinosis.

**Figure 1:**
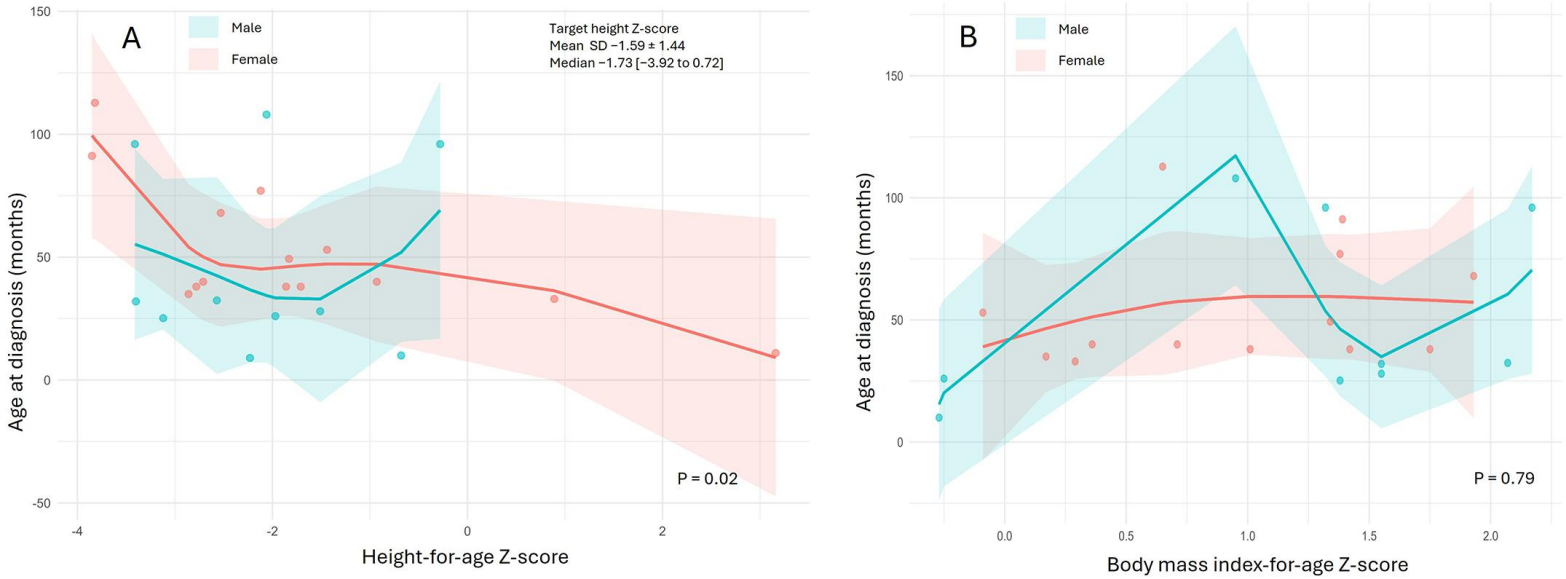
Baseline anthropometric characteristics according to age at diagnosis in patients with X-linked hypophosphatemia. **(A)** Relationship between height-for-age Z-score and age at diagnosis. The vertical dashed line indicates the mean target height Z-score. **(B)** Relationship between BMI-for-age Z-score and age at diagnosis. Symbols represent individual patients stratified by sex. Solid lines represent locally weighted scatterplot smoothing (LOESS) curves, and shaded areas indicate 95% confidence intervals.

At diagnosis, the mean height-for-age Z-score was −1.90 ± 1.56 (*n* = 41), reflecting a marked baseline growth deficit (Supplementary Table S1). An inverse correlation was observed between height-for-age Z-score at diagnosis and age at diagnosis (*r* = −0.77; *P* = 0.02). The LOESS smoothing curve demonstrated a progressive decline in height-for-age Z-score with increasing age at diagnosis, indicating greater growth impairment among patients diagnosed later (Figure 1A). In contrast, no significant correlation was observed between BMI-for-age Z-score at diagnosis and age at diagnosis (Figure 1B; *P* = 0.79), and the corresponding LOESS curve showed no consistent trend across the age range. When stratified by sex, height-for-age Z-scores were similar between males and females, with no significant difference observed (*P* = 0.79) (Table 1).

**Table 1:** Baseline characteristics stratified by sex.

Characteristics at diagnosis (*n* = 41)	Sex		
	Female 63.41% (*n* = 26)	Male 36.59% (*n* = 15)	*P*-value
Height-for-age Z-score ^1^	−2 [−2.8–−1.5]	−2.1 [−3–−1.6]	0.79
BMI-for-age Z-score^2^	0.9 ± 0.6	1.2 ± 0.9	0.54
Target height Z-score^2^	−1.5 ± 1.6	−1.8 ± 1.1	0.68
Radiographic abnormalities, *n* (%)^3^	23 (88.5)	13 (86.7)	0.60
Lower limb deformities, *n* (%)^3^	14 (53.8)	10 (66.7)	0.51

1 Values are expressed as median (25th–75th percentiles). Comparisons were performed using the Mann–Whitney *U*-test. 2 Values are expressed as mean ± standard deviation. Comparisons were performed using the Student's *t*-test. All measurements were transformed into z-scores by comparing them with age- and sex-specific norms for healthy children.3 Fisher's exact test.

Of the 41 patients, 38 (92.7%) received conventional therapy and three (7.3%) received burosumab. Among patients with XLH and lower limb deformities, 57.7% (24/41) underwent orthopedic surgery. Radiographic abnormalities at diagnosis were highly prevalent in both sexes, being observed in 88.5% of females and 86.7% of males, with no significant difference between groups (*P* = 0.60). Similarly, lower limb deformities were present in 53.8% of females and 66.7% of males, with comparable frequencies between sexes (*P* = 0.51) (Table 1). No significant association was observed between sex and the need for surgical intervention (*P* = 0.09). All surgeries were performed after age 6 years and only after metabolic stabilization.

**Figure 2:**
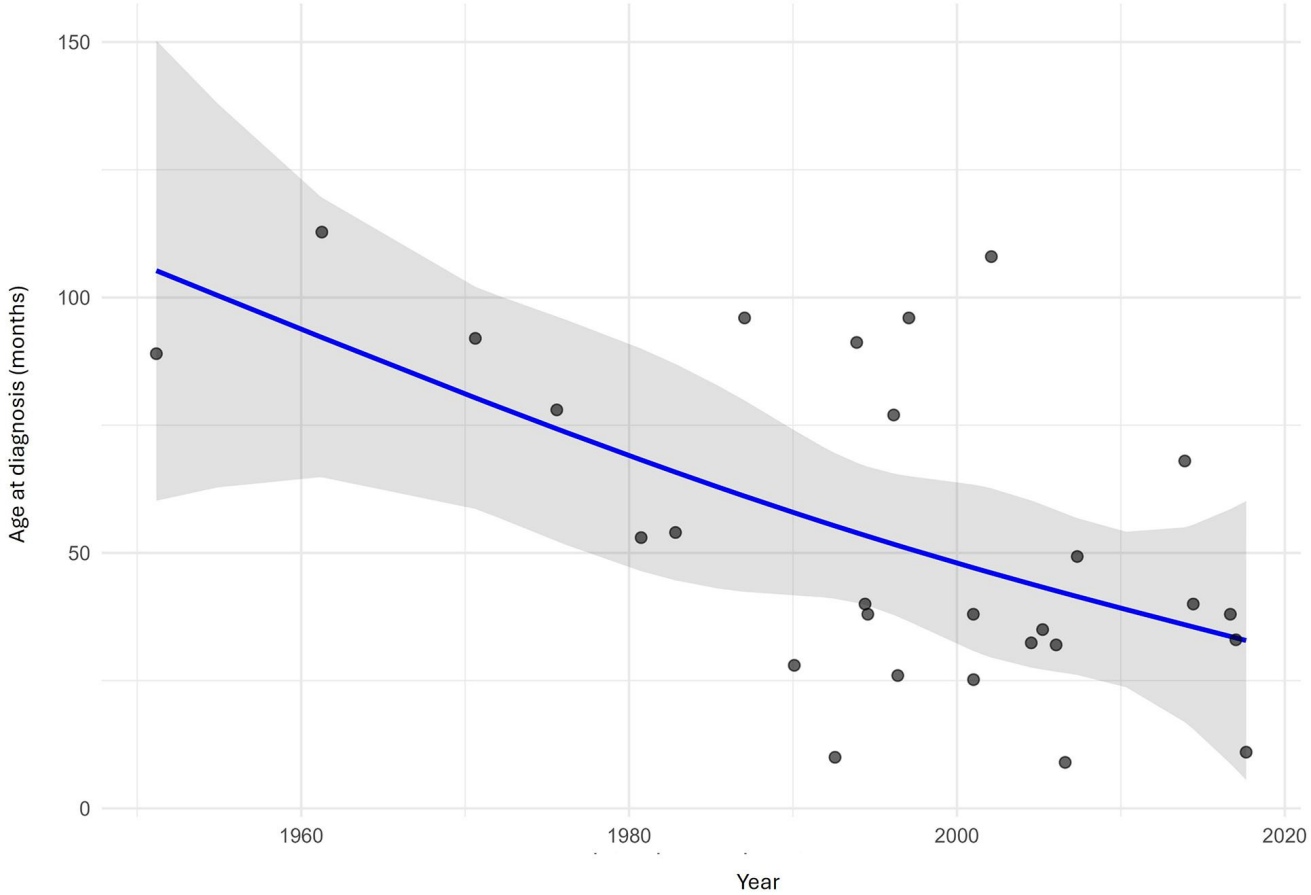
Temporal trends in age at diagnosis among patients with X-linked hypophosphatemia. Each point represents an individual patient. The solid blue line represents the fitted linear regression model, and the shaded area indicates the 95% confidence interval. Age at diagnosis decreased significantly over successive birth cohorts (*β* = −1.1 months/year; 95% CI, −1.7 to −0.4; *P* = 0.002).

Age at diagnosis decreased significantly across successive birth cohorts. Linear regression analysis demonstrated a reduction of approximately 1.1 months in age at diagnosis for each additional year of birth (*β* = −1.1 months/year; 95% CI, −1.7 to −0.4; *P* = 0.002) (Figure 2).

### Growth outcomes and determinants of final height

Final height was achieved in 20 patients treated exclusively with conventional therapy, who were included in the analysis of growth outcomes, whereas three patients exposed to burosumab were excluded from this analysis. Family history was present in 13/20 patients (65.0%), including affected offspring (6/20; 30.0%), parents (3/20; 15.0%), and extended relatives (4/20; 20.0%), while 7/20 (35.0%) were classified as index cases without a known family history, consistent with X-linked dominant inheritance.

Genetic analysis revealed a heterogeneous spectrum of PHEX variants, including nonsense (6/20; 30.0%), missense (6/20; 30.0%), frameshift (3/20; 15.0%), splice site (2/20; 10.0%), exon deletions (2/20; 10.0%), and intronic variants (1/20; 5.0%), all predicted to be deleterious by in silico analysis (Supplementary Table S2).

**Figure 3:**
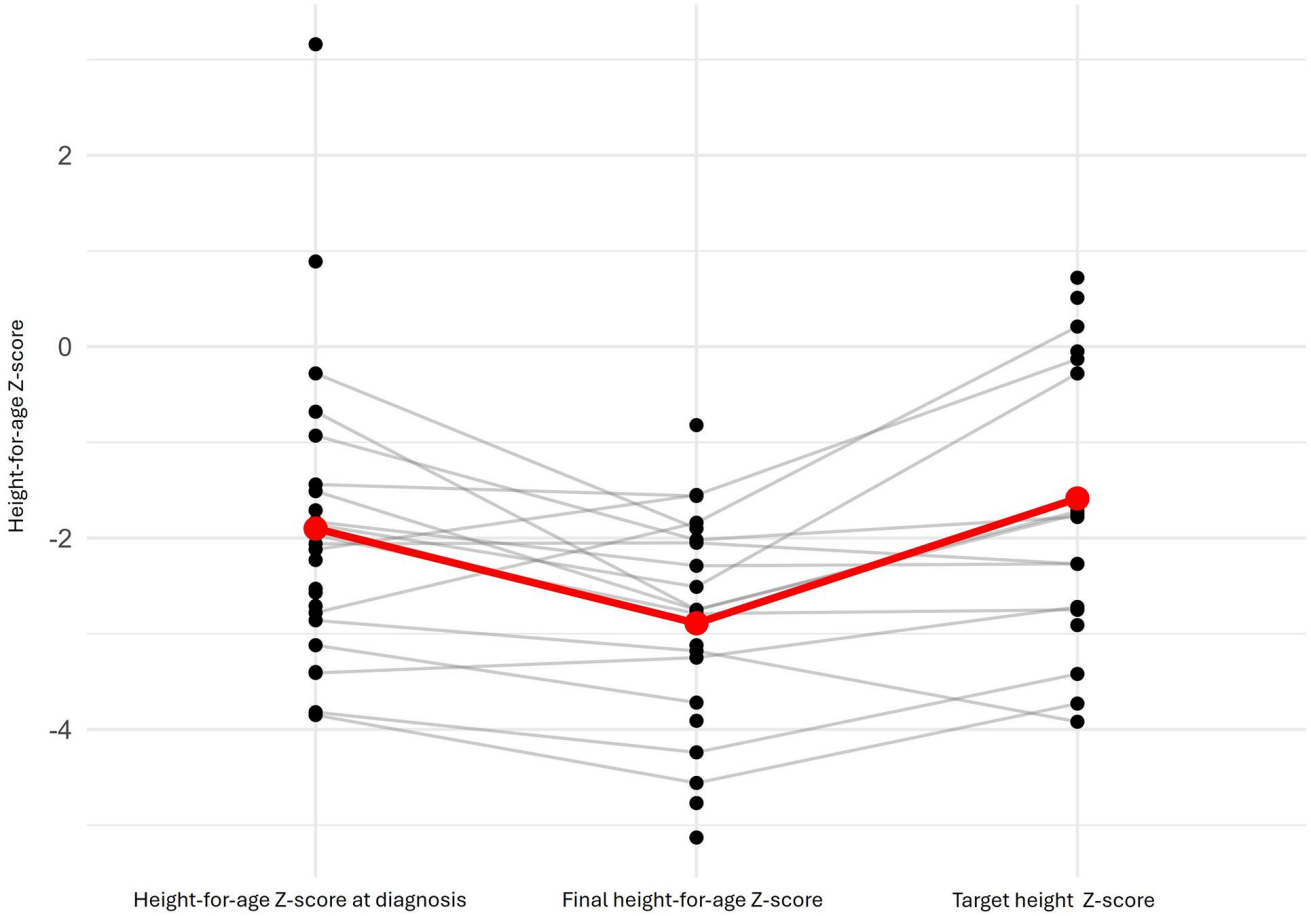
Height-for-age Z-score at diagnosis, final height Z-score, and target height Z-score among patients with X-linked hypophosphatemia treated exclusively with conventional therapy (*n* = 20). Black dots represent individual patients and gray lines connect measurements from the same patient. Red circles and connecting lines represent median values for each variable.

Among the 20 patients who reached final height and were treated exclusively with conventional therapy, the mean age at diagnosis was 56.28 ± 33.19 months. At diagnosis, the mean height-for-age Z-score was −2.16 ± 1.11, while the mean target height Z-score was −2.20 ± 1.26 (Supplementary Table S3). At final height, the mean height-for-age Z-score was −2.96 ± 1.15, indicating persistent short stature (Supplementary Table S3). Figure 3 illustrates the individual trajectories from height-for-age Z-score at diagnosis to final height Z-score and target height Z-score, highlighting substantial variability in growth outcomes across the cohort.

In multivariable linear regression analysis, height-for-age Z-score at diagnosis (*β* = 0.10; 95% CI 0.04 to 0.15; *P* = 0.009), age at diagnosis (*β* = −0.04; 95% CI −0.07 to −0.02; *P* = 0.007), and target height Z-score (*β* = 0.37; 95% CI 0.15 to 0.59; *P* = 0.01) were independently associated with final height-for-age Z- score. In contrast, sex (*β* = 0.30; 95% CI −0.18 to 0.79; *P* = 0.25) and BMI-for-age Z score (*β* = 0.19; 95% CI −0.10 to 0.56; *P* = 0.38) were not significantly associated with the outcome (Table 2).

**Table 2:** Multivariable linear regression analysis of factors associated with final height-for-age Z-score (*n* = 20).

Variable	*β* coefficient (95% CI)	*P* value
Intercept	−8.78 (−12.73 to −4.84)	0.003
Height-for-age Z-score	0.10 (0.04 to 0.15)	0.009
Age at diagnosis (months)	−0.04 (−0.07 to −0.02)	0.007
Target height Z-score	0.37 (0.15 to 0.59)	0.01
Sex	0.30 (−0.18 to 0.79)	0.25
BMI-for-age Z-score	0.19 (−0.10 to 0.56)	0.38

β coefficients represent adjusted estimates from the multivariable linear regression model. Model performance was assessed using the coefficient of determination (R²). CI = confidence interval. BMI Body mass index.

## Discussion

X-linked hypophosphatemia is a rare disorder with substantial impact on growth and skeletal development, particularly when diagnosis is delayed. In real-world settings such as the Brazilian public health system, access to specialized care may occur later in the disease course, potentially influencing clinical presentation at diagnosis. In this cohort of patients followed at a tertiary referral outpatient clinic, we observed a significant inverse relationship between age at diagnosis and baseline height Z score, indicating that delayed recognition was associated with more severe growth impairment at presentation.

These findings are consistent with prior reports demonstrating that growth deficit in XLH begins early in life and progresses during childhood. Mäkitie et al (19). showed that children with XLH typically have normal length at birth but develop progressive growth retardation in the first years of life, even under conventional therapy. Although pubertal growth during conventional treatment has been reported to be relatively preserved in patients with XLH, it is generally insufficient to compensate for the growth deficits established during early childhood. Consequently, a substantial proportion of the final height deficit appears to be determined before diagnosis and initiation of treatment (19). Taken together, these observations support the concept that early childhood represents a critical period for growth in XLH, during which delays in diagnosis may result in irreversible impairment of final stature.

In contrast to height, no significant association was observed between age at diagnosis and BMI Z score at presentation. This result is consistent with the pathophysiology of XLH, in which linear growth is directly affected by chronic hypophosphatemia, whereas BMI reflects a more complex interplay between body composition, physical activity, and long-term disease burden (20). Previous studies have shown an increased prevalence of overweight and obesity in patients with XLH, likely related to impaired mobility and altered biomechanics, rather than early disease severity (20, 21). Together, these observations suggest that, unlike height, BMI at diagnosis is not a sensitive marker of disease duration or timing of diagnosis.

No significant differences were observed between male and female patients in baseline height Z score, BMI Z score, or familial target height. Although previous studies have suggested that males may exhibit more severe phenotypes because of hemizygosity for PHEX variants, we did not observe significant sex related differences in baseline anthropometric characteristics or orthopedic intervention rates in our cohort. These findings highlight the substantial phenotypic variability of XLH and suggest that factors other than sex may play a more prominent role in determining disease presentation at diagnosis (1, 6, 22). These findings suggest that, at presentation, growth impairment may be influenced more by timing of diagnosis than by sex related biological differences.

Delayed diagnosis remains a major challenge in rare diseases, particularly in middle income countries such as Brazil, where patients often face prolonged diagnostic pathways within the public health system (23). Although the Brazilian National Policy for Rare Diseases was established in 2014, aiming to improve access to diagnosis and multidisciplinary care, its implementation remains heterogeneous across the country, given its continental dimensions and regional disparities in healthcare infrastructure (24). In this context, our findings provide specific evidence that delayed diagnosis in XLH is directly associated with greater growth impairment at presentation, reinforcing the importance of early recognition, timely referral to specialized centers, and expansion of diagnostic capacity within the public health system.

In this study, familial target height was independently associated with final height in multivariable analysis, indicating that genetic growth potential remains a major determinant of adult stature in patients with XLH despite chronic phosphate wasting. In addition, height-for-age Z-score at diagnosis and age at diagnosis were independently associated with final height, reinforcing the combined influence of baseline growth impairment and timing of diagnosis on long-term outcomes. These findings are consistent with previous studies demonstrating that growth failure in XLH begins early in life, progresses during childhood, and is only partially corrected with conventional therapy, resulting in persistently reduced adult height (25). Furthermore, substantial phenotypic variability persists even among individuals with similar genetic variants, supporting the concept that final height reflects a complex interplay between disease-related factors and intrinsic growth potential (26). This interpretation is further supported by historical cohorts evaluating long-term growth outcomes in XLH. Miyamoto et al. reported that although treatment with phosphate and active vitamin D improved growth, final height remained below expected population norms in a cohort of Japanese patients with XLH (27). Similarly, Chesher et al., in a cohort of molecularly confirmed adults with PHEX mutations, documented persistent short stature and substantial skeletal morbidity despite long-term treatment (28). Taken together, these findings and our results reinforce that conventional therapy only partially mitigates the growth impairment associated with XLH and that adult height remains significantly reduced in many patients, particularly when diagnosis and treatment are delayed.

This study has several limitations. Its retrospective design and extended study period, spanning multiple decades, may have introduced heterogeneity in diagnostic practices and clinical management over time. The number of patients who reached final height was relatively small, which may limit the statistical power of subgroup analyses and increase the risk of overfitting in multivariable models. To mitigate this, the regression analysis was restricted to a limited number of clinically relevant variables selected *a priori*; however, the findings should be interpreted as exploratory rather than confirmatory. In addition, exposure to burosumab was limited to more recent cases (*n* = 3) and not included in the final height analysis, precluding assessment of its long-term effects. Referral to a tertiary center may have introduced selection bias toward more severe phenotypes. Furthermore, the use of historical data may have resulted in incomplete information for some variables, although efforts were made to ensure data consistency.

Despite these limitations, this study has important strengths. It represents one of the longest observational cohorts of patients with XLH, with follow-up extending from 1971 to 2025. All cases were molecularly confirmed, ensuring diagnostic accuracy, and anthropometric measurements were obtained using standardized methods. Moreover, the study provides real-world data from a Brazilian cohort, offering relevant insights into disease presentation and management in a middle-income setting, where delayed diagnosis remains a critical challenge.

In conclusion, final height in XLH is determined by a complex interplay between genetic potential, baseline growth impairment, and timing of diagnosis. Our findings demonstrate that delayed diagnosis is associated with more severe growth deficits at presentation and contributes to suboptimal adult height, even under conventional therapy. The strong association between familial target height and final height further highlights the persistent influence of genetic determinants despite chronic disease. These results underscore the importance of early recognition and timely referral to specialized centers, particularly in healthcare settings where diagnostic delays remain common. Moreover, this cohort provides a relevant benchmark for growth outcomes in the pre-burosumab era, offering a foundation for future comparisons with targeted therapies aimed at improving long-term skeletal outcomes in patients with XLH.

## Data Availability

The raw data supporting the conclusions of this article will be made available by the authors, without undue reservation.
