# Supplementary material for: Determinants of final height in X-linked hypophosphatemia: impact of diagnostic delay and baseline growth in a Brazilian cohort
Source: Front Pediatr. 2026 Jul 2;14:1845607. doi: 10.3389/fped.2026.1845607 (PMC13372968; doi:10.3389/fped.2026.1845607)
Supplement: Supplementary file 1 [file Supplementaryfile1.docx]

**Determinants of Final Height in X-Linked Hypophosphatemia: Impact of Diagnostic Delay and Baseline Growth in a Brazilian Cohort**

**Supplementary Material**

**Borghi, et al.**

**Supplementary Table 1.** Baseline characteristics and anthropometric outcomes at diagnosis.

| **Characteristics at diagnosis (n = 41)** | Mean | SD | Median | First quartile (Q1) | Third quartile (Q3) | Minimum | Maximum |
| --- | --- | --- | --- | --- | --- | --- | --- |
| Age (months) | 49.64 | 30.67 | 38.00 | 32.00 | 68.00 | 9.00 | 112.80 |
| height-for-age Z-score | -1.90 | 1.56 | -2.09 | -2.80 | -1.49 | -3.85 | -3.16 |
| BMI-for-age Z-score | 1.04 | 0.74 | 1.33 | 0.43 | 1.52 | -0.27 | 2.17 |
| Target height Z-score | -1.59 | 1.44 | -1.73 | -2.73 | -0.24 | -3.92 | 0.72 |

All measurements were transformed into z-scores by comparing them with age- and sex-specific norms for healthy children.

**Supplementary Table 2**. Genetic characteristics of *PHEX* variants in patients who reached final height.

| **cDNA mutation^1^** | **Classification** | **Variant site** | **In silico prediction^2^** |
| --- | --- | --- | --- |
| c.1645C>T | Nonsense | 15 | deleterious |
| c.1582_1583delAC | Frameshift | 14 | deleterious |
| c.422C>T | Missense | 4 | deleterious |
| c.422C>T | Missense | 4 | deleterious |
| c.1645+1G>A | Splice site variant | 15 | deleterious |
|  | Deletion of exon 18 | 18 | deleterious |
| c.1779T>A | Nonsense | 18 | deleterious |
| c.985delC | Frameshift | 9 | deleterious |
| c.2104C>T | Nonsense | 21 | deleterious |
| c.1699C>T | Nonsense | 15 | deleterious |
| c.1645C>T | Nonsense | 15 | deleterious |
| c.422C>T | Missense | 4 | deleterious |
| c.1862A>C | Missense | 18 | deleterious |
|  | Deletion of exons 13 to 15 | 13-15 | deleterious |
| c.1645C>T | Nonsense | 15 | deleterious |
| c.1862A>C | Missense | 18 | deleterious |
| c.1862A>C | Missense | 18 | deleterious |
| c.1173+5G>A | Variant located in an intronic region | 19 | deleterious |
| c.1769-2A>G | Splice site variant | 17 | deleterious |
| c.70_74delGTCGT | Frameshift | 1 | deleterious |

^1^Abbreviation: cDNA, complementary DNA (Ref Seq: NM_000444.6). ^2^In silico predictors used - Missenses: SIFT, MutPred, MutationTaster, CAD-score variants; Splice-site: Mutation taster, splice AI and Human Splicing Finder; Frameshift and nonsense: Mutation Taster and CAD-score variants.

**Supplementary Table 3.** Baseline and final anthropometric characteristics of patients who reached final height (n = 20).

| **Characteristics at diagnosis (n = 20)** | Mean | SD | Median | First quartile (Q1) | Third quartile (Q3) | Minimum | Maximum |
| --- | --- | --- | --- | --- | --- | --- | --- |
| Age (months) | 56.28 | 33.19 | 44.65 | 33.25 | 92.40 | 10.00 | 112.80 |
| Height-for-age Z-score | -2.16 | 1.11 | -1.97 | -2.99 | -1.48 | -3.85 | -0.28 |
| BMI-for-age Z-score | 0.90 | 0.76 | 1.01 | 0.26 | 1.38 | -0.27 | 2.17 |
| Target height Z-score | -2.20 | 1.26 | -2.27 | -2.92 | -1.74 | -3.92 | 0.21 |
| **Anthropometric outcomes at final height (n = 20)** | Mean | SD | Median | First quartile (Q1) | Third quartile (Q3) | Minimum | Maximum |
| Final height-for-age Z-score | -2.96 | 1.15 | -2.77 | -3.77 | -2.04 | -5.13 | -0.82 |
| Final BMI-for-age Z-score | 1.32 | 0.75 | 1.13 | 0.80 | 2.01 | -0.19 | 2.50 |

All measurements were transformed into z-scores by comparing them with age- and sex-specific norms for healthy children.
